# Percutaneous mechanical thrombectomy versus catheter-directed thrombolysis for the treatment of arterial acute mesenteric ischemia and risk factors for 30-day mortality

**DOI:** 10.3389/fcvm.2025.1553170

**Published:** 2025-05-16

**Authors:** Yadong Shi, Yangyi Zhou, Hao Huang, Liang Chen, Haobo Su, Jianping Gu

**Affiliations:** Department of Vascular and Interventional Radiology, Nanjing First Hospital, Nanjing Medical University, Nanjing, China

**Keywords:** acute mesenteric ischemia, endovascular revascularization, catheter-directed thrombolysis, risk factors, 30-day mortality, percutaneous mechanical thrombectomy

## Abstract

**Objective:**

To compare the efficacy and safety outcomes between percutaneous mechanical thrombectomy (PMT) and catheter-directed thrombolysis (CDT) as the first endovascular revascularization (EVR) strategy for arterial acute mesenteric ischemia (AMI) and identify risk factors for 30-day mortality.

**Methods:**

This was a single-center retrospective study. Between May 2014 and March 2024, consecutive patients with arterial AMI who received EVR using PMT or CDT as the first strategy were included. The baseline characteristics, imaging information, procedure-related information, complications, and clinical outcomes of patients were analyzed and compared. Binary logistic regression analysis was used to identify potential risk factors for 30-day mortality with an odds ratio (OR) and 95% confidence interval (CI).

**Results:**

Forty-seven patients (PMT, *n* = 29; CDT, *n* = 18) were included. The mean age was 74.3 ± 7.6 years, and 66.0% were female. Successful revascularization was achieved in 89.4% of patients, and the 30-day mortality rate was 31.9%. There was no significant difference in successful revascularization, complications, and clinical outcomes between PMT and CDT as the first strategy. High plasma lactate (adjusted OR 1.73 per 1.0 mmol/L increase, 95% CI: 1.13–2.66; *p* = 0.012) and D-dimer (adjusted OR 1.73 per 1.0 mg/L increase; 95% CI: 1.20–2.50; *p* = .003) were associated with a high 30-day mortality rate.

**Conclusions:**

PMT and CDT were associated with high revascularization rates and few complications. High plasma lactate and D-dimer may be associated with high 30-day mortality.

## Introduction

Acute mesenteric ischemia (AMI) is considered an uncommon source of abdominal pain, and the incidence is approximately 6/100,000 person-years (1). Despite the development of treatment in the last decades, AMI remains a lethal scenario with short-term mortality of over 50% (1–3), and arterial AMI is the most lethal scenario among all subtypes (2).

Compared with Open surgical revascularization (OSR), endovascular revascularization (EVR) is characterized as minimally invasive and may be more appropriate for elderly and frail patients (4). Moreover, EVR has shown superiorities in improved clinical outcomes and cost-effectiveness (5–7). As such, EVR techniques have been increasingly advocated as the first strategy in recent years (8, 9). Various EVR techniques, including percutaneous mechanical thrombectomy (PMT), catheter-directed thrombolysis (CDT), percutaneous transluminal angioplasty (PTA), and stent placement, have been introduced in the treatment of arterial AMI depending on the cause of occlusion (10), but the optimal EVR technique for arterial AMI remains undetermined (11).

The present study aimed to investigate the differences regarding the efficacy and safety between PMT and CDT as the first EVR technique for the treatment of arterial occlusive AMI. In addition, the potential risk factors for 30-day mortality were evaluated.

## Methods

### Study design

This was a single-center retrospective observational study. Between May 2014 and March 2024, consecutive patients with computed tomography angiography (CTA) confirmed arterial occlusive AMI who received EVR using either PMT or CDT as the first EVR technique were included. Patients who underwent PMT as the first technique and required adjunctive CDT therapy were included in the PMT group. The patients who received direct stent placement for revascularization were excluded. In the study center, EVR was performed as the first-line revascularization treatment for arterial AMI patients. Two researchers independently searched the electronic medical recording system to identify potential candidates using the International Classification of Diseases 10th version (ICD-10) code K55.0.

Patient demographics, duration of symptom onset to diagnosis, etiology of arterial AMI, manifestations, comorbidities, and pre-procedure laboratory tests were collected and compared. The pre-procedure imaging information was reviewed. Procedure-related information, initial and final revascularization efficacy, complications, and clinical outcomes were noted and compared. The study was conducted in accordance with the Declaration of Helsinki. For patients who underwent stent thrombectomy, explicit written consent for the off-label use of the stent retriever was obtained from patients or their relatives before the procedures. The Institutional Review Board of the study hospital approved this study protocol, and informed consent was waived due to the retrospective study design.

### Revascularization procedure and post-procedure management

In the present study, both PMT (including manual aspiration and stent thrombectomy) and CDT were performed as the first EVR techniques. The decision of the different first EVR techniques was predominantly made by the operators based on the severity of symptoms, anatomic locations of occlusion, and experiences. For instance, patients with milder symptoms and distal occlusion with a small SMA diameter may receive CDT first. Patients with severe symptoms and target vessel diameter suitable for PMT devices may receive PMT first.

Previous studies have described the detailed process for manual aspiration and stent thrombectomy (12, 13). In summary, for patients who received manual aspiration, a 6-Fr guiding sheath (Super Arrow-Flex; Teleflex, USA) was initially placed in the superior mesenteric artery (SMA), and then a 6-Fr aspiration catheter (Mach 1 Peripheral Guide Catheter; Boston Scientific, USA) was advanced into the thrombus gently, and aspiration was performed using a 60 ml syringe. For patients who underwent stent thrombectomy, a 6-Fr guiding sheath (Super Arrow-Flex) was initially placed in the SMA. A 2.8-Fr microcatheter (Progreat; Terumo, Japan) and a 6-Fr guiding catheter (Mach 1 Peripheral Guide Catheter) were introduced coaxially. After successfully crossing the occlusion, a Solitaire AB stent (Medtronic, USA) was deployed across the thrombus. After sufficient stent expansion, the stent was retrieved through the guiding catheter, and manual aspiration using the guiding catheter was synchronously performed. The Rotarex (Straub Medical, Switzerland) thrombectomy was performed as the previously described approach (14).

CDT was performed as the first EVR technique or as an adjunctive procedure in patients with residual thrombus after the initial PMT procedure. CDT was also performed for the residual branch vessel involvement that notably compromised distal infusion. A 4-Fr infusion catheter (UniFuse; AngioDynamics, USA; or Fountain; Merit Medical Systems, USA) or an end-hole catheter was placed in the target vessel. Recombinant tissue plasminogen activator (Actilyse; Boehringer Ingelheim, Germany) was continuously infused at 10.0–20.0 mg/24 h (15). Follow-up angiography was performed every 12–24 h.

No additional endovascular treatment was performed for distal embolization as this occlusion rarely deteriorated perfusion of the intestine owing to the abundant mesenteric collateral network (16).

PTA and stent placement were performed to correct coexisting atherosclerotic stenosis >50%. A self-expandable stent (EverFlex; Medtronic, USA; or Smart Control; Cordis, USA) was used in the present study.

After the revascularization procedure, patients were monitored for any complications, potential symptom aggravation, or signs of bowel necrosis. Patients received bowel rest, parenteral nutritional support, pain management, antithrombotic therapy, and intravenous broad-spectrum antibiotics (17). If patients exhibited pre-procedure and post-procedure signs of bowel necrosis, laparotomy with resection of the necrotic bowel was performed as needed. The decision to perform a second-look laparotomy is made based on the surgeon's interpretation of the initial laparotomy.

For embolic AMI, anticoagulation therapy with low-molecular weight heparin, rivaroxaban, or warfarin was prescribed based on per patient's condition. Lifelong anticoagulation was recommended if there were no contraindications. For thrombotic AMI, antiplatelet therapy with aspirin and/or clopidogrel was prescribed (4).

### Definitions

Embolic occlusion was defined as a thrombus surrounded by contrast material in a noncalcified SMA in conjunction with the acute onset of symptoms (18). Thrombotic occlusion was defined as a thrombotic occlusion in a calcified or stenosis SMA (18). Complete revascularization was defined as complete SMA stem recanalization without residual thrombus on angiography. Partial revascularization was defined as the partial SMA stem recanalization with residual thrombus but not significantly compromising distal vessel opacification on angiography. Successful revascularization was defined as both complete and partial revascularization. Failed revascularization was defined as the inability to remove the thrombus after treatment. Short bowel syndrome was defined as the need for parenteral nutrition support (19). Proximal SMA was defined as the segment between the ostium and the origin of the inferior pancreaticoduodenal artery. Middle SMA was defined as the segment between the pancreaticoduodenal and the ileocolic artery. Distal SMA was defined as the vascular bed downstream from the ileocolic artery (20). The signs suggested bowel necrosis include persistent peritonitis, pneumatosis intestinalis, portomesenteric venous gas, and free peritoneal gas (21).

### Statistical analysis

Data were processed and analyzed using the SPSS statistical package (26.0; IBM SPSS Statistics, USA). The distribution of continuous data was tested using the Shapiro–Wilk test. Normally distributed data were presented as mean ± standard deviation, and asymmetrically distributed data were presented as the median and interquartile range (IQR). The Student *t*-test or Manne–Whitney *U* test was performed to compare the difference between continuous data, and the chi-square test or Fisher's exact test was used for categorical data where appropriate. The potential risk factors for 30-day mortality were initially identified using univariable analysis and subsequently estimated using the logistic regression models with an odds ratio (OR) and 95% confidence interval (CI). A two-tailed *p*-value <.050 was considered statistically significant.

## Results

### Patients

**Figure 1:**
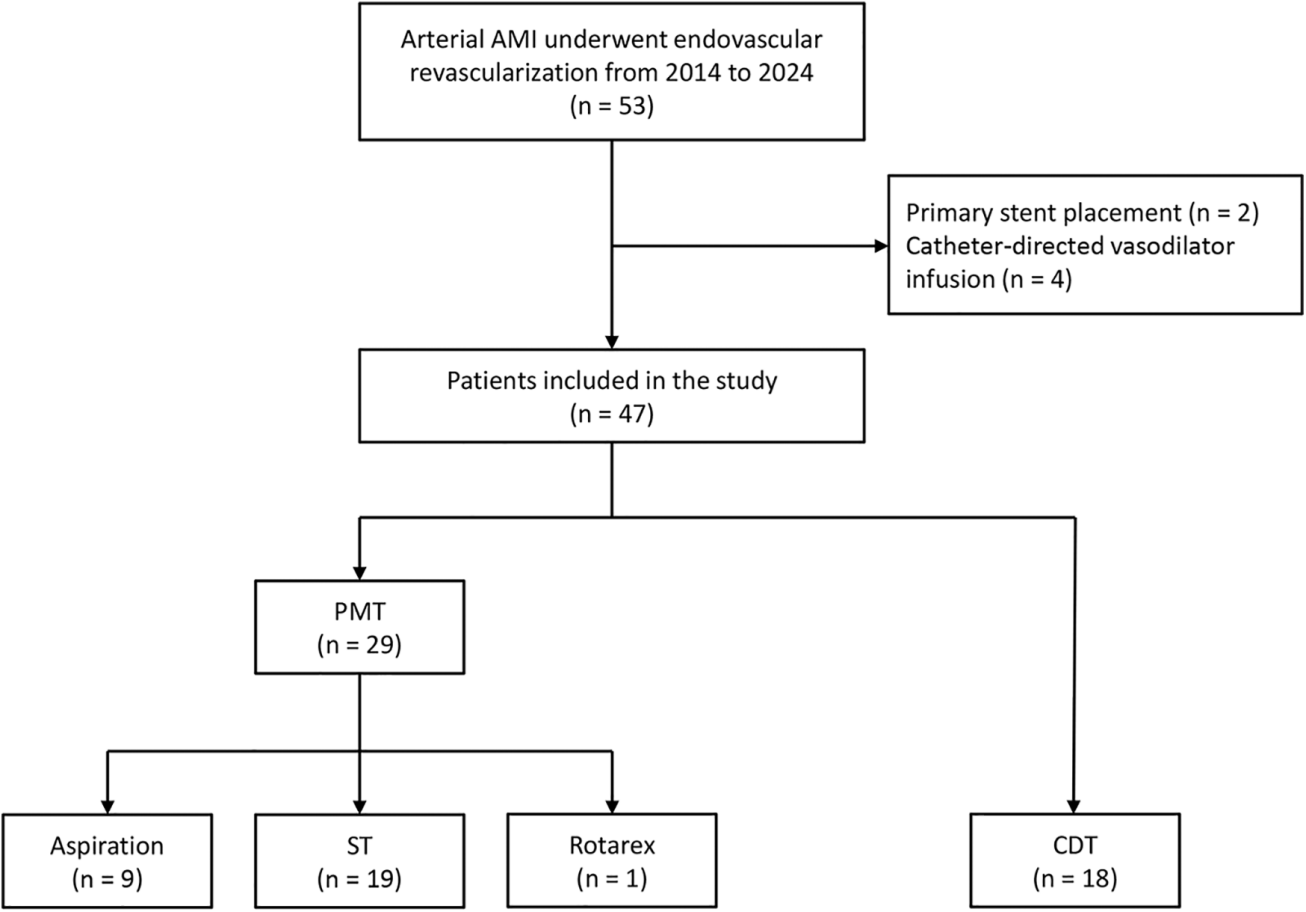
The flowchart of patients with acute mesenteric ischemia inclusion and first endovascular revascularization choice.

During the study period, 47 patients with arterial occlusive AMI were included. Embolic and thrombotic occlusion accounted for 80.9% and 19.1% of the patients. The mean age was 74.3 ± 7.6 years, and 66.0% were female. Figure 1 presents the detailed patient inclusion flowchart and the first EVR technique choice.

The median duration from symptom onset to admission was 15.0 h (IQR: 8.0–24.0 h). Thrombotic and embolic occlusion etiology accounted for 19.1% and 80.9% of patients, respectively. All patients had acute abdominal pain, and other common manifestations included diarrhea (42.6%) and vomiting (29.8%). The median symptom duration in the PMT group was significantly lower than in the CDT group (10.0 h vs. 24.0 h, *p* = .003). The main comorbidities were hypertension (64.8%) and atrial fibrillation (59.6%). Pre-procedure laboratory tests found notable elevated white blood cell count and D-dimer value. SMA occlusion since proximal, middle, and distal SMA was noted in 10 (21.3%), 29 (61.7%), and 8 (17.0%) patients, respectively. There was no significant difference regarding occlusion location between PMT and CDT groups (Table 1). SMA branch involvement, synchronous embolization, and evidence of bowel necrosis were noted in 12.8%, 6.4%, and 10.6% of patients, respectively.

**Table 1:** Comparison of baseline characteristics and image findings of 47 patients with arterial acute mesenteric ischemia underwent PMT and CDT as primary revascularization.

Variable	Overall (*n* = 47)	PMT (*n* = 29)	CDT (*n* = 18)	*p* value
Demographics				
Mean age – year	74.3 ± 7.6	75.0 ± 7.1	73.3 ± 8.5	0.46
Female	31 (66.0%)	21 (72.4%)	10 (55.6%)	0.17
Symptom to admission—hour	15.0 (8.0–24.0)	10.0 (6.0–19.0)	24.0 (12.0–26.5)	0.003
Etiology				
Thrombotic occlusion	9 (19.1%)	4 (13.8%)	5 (27.8%)	
Embolic occlusion	38 (80.9%)	25 (86.2%)	13 (72.2%)	0.27
Manifestations				
Abdominal pain	47 (100%)	29 (100%)	18 (100%)	–
Nausea	10 (21.3%)	7 (24.1%)	3 (16.7%)	0.72
Vomiting	14 (29.8%)	9 (31.0%)	5 (27.8%)	0.81
Diarrhea	20 (42.6%)	13 (44.8%)	7 (38.9%)	0.69
Hematochezia	9 (18.4%)	8 (27.6%)	1 (5.6%)	0.065
Peritonitis	3 (6.4%)	2 (6.9%)	1 (5.6%)	1.00
Comorbidities				
Hypertension	30 (64.8%)	18 (62.1%)	12 (66.7%)	0.75
Diabetes	11 (23.4%)	6 (20.7%)	5 (27.8%)	0.73
Atrial fibrillation	28 (59.6%)	20 (69.0%)	8 (44.4%)	0.10
Rheumatic heart disease	3 (6.4%)	1 (3.4%)	2 (11.1%)	0.55
Coronary artery diseases	11 (23.4%)	5 (17.2%)	6 (33.3%)	0.29
Cardiac insufficiency	7 (14.9%)	5 (17.2%)	2 (11.1%)	0.69
Ischemic stroke	13 (27.7%)	7 (24.1%)	6 (33.3%)	0.52
Renal insufficiency	5 (10.6%)	3 (10.3%)	2 (11.1%)	1.00
Pre-procedure laboratory tests				
White blood cell count—10^9^/L	15.4 (9.7–18.8)	15.3 (9.6–16.9)	15.5 (11.0–22.3)	0.61
Plasma creatinine—μmol/L	96.3 (62.7–123.9)	100 (61.0–137.5)	84.0 (62.6–105.0)	0.38
Plasma lactate—mmol/L	1.9 (1.2–4.2)	2.4 (1.2–4.2)	1.7 (1.2–4.5)	0.070
D-dimer—mg/L	3.1 (1.7–5.4)	3.5 (1.8–7.2)	2.7 (1.2–3.8)	0.96
Image findings				
Occlusion initiation location				
Proximal SMA	10 (21.3%)	6 (20.7%)	4 (22.2%)	
Middle SMA	29 (61.7%)	20 (69.0%)	9 (50.0%)	
Distal SMA	8 (17.0%)	3 (10.3%)	5 (27.8%)	0.27
SMA branch involvement	6 (12.8%)	4 (13.8%)	2 (11.1%)	1.00
Synchronous embolization	3 (6.4%)	1 (3.4%)	2 (11.1%)	0.55
Evidence of bowel necrosis	5 (10.6%)	2 (6.9%)	3 (16.7%)	0.36

Data are presented with *n* (%) or median (interquartile range) unless otherwise stated.

SMA, superior mesenteric artery; PMT, percutaneous mechanical thrombectomy; CDT, catheter-directed thrombolysis.

### Clinical outcomes and comparison between PMT and CDT as the first technique

Adjunctive PTA and stent placement were required in 10.6% and 10.6% of patients, respectively (Table 2). Finally, successful revascularization was achieved in 89.3% of patients. Five patients encountered unsuccessful revascularization due to the following reasons: 3 patients had multiple revascularization attempts but still failed, and the patients were unable to tolerate the subsequent attempts; 2 patients encountered severe vessel dissection during revascularization. All patients who experienced unsuccessful EVR refused open surgical revascularization, and they all died at 30-day follow-up. The overall procedure-related complications, including dissection, new distal embolization, and bleeding events, were encountered in 4.3%, 8.5%, and 4.3% of patients, respectively. The two bleeding events were minor, with hematoma around the sheath, and the bleeding events were successfully managed with manual compression and thrombolysis cessation. The overall bowel resection and second-look laparotomy was required in 12.8% and 14.9% of the patients, respectively. The median length of hospital stay was 7.0 days (IQR: 2.0–11.0 days). The overall in-hospital mortality and 30-day mortality were 10.6% (5/47) and 31.9% (15/47), respectively. The reasons for death were multiple organ dysfunction syndrome (80%) and septic shock (20%). Short bowel syndrome was noted in 16.7% (1/6) of surviving patients who received bowel resection at 30-day follow-up. No recurrent thrombosis or readmission was noted at the 30-day follow-up.

**Table 2:** Outcomes of 47 patients underwent PMT and CDT as the primary revascularization for arterial AMI.

Variable	Overall (*n* = 47)	PMT (*n* = 29)	CDT (*n* = 18)	*p* value
Vascular access				
Femoral	46 (97.9%)	28 (96.6%)	18 (100%)	
Brachial	1 (2.1%)	1 (3.4%)	0	1.00
Adjunctive treatment				
Local thrombolysis	–	7 (24.1%)	–	
Thrombolytic agent dosage—mg	–	10.0 (10.0–20.0)	20.0 (13.8–30)	0.029
Thrombolysis duration—day	–	1.0 (1.0–2.0)	2.5 (2.0–3.3)	0.027
PTA	5 (10.6%)	3 (10.3%)	2 (11.1%)	1.00
Stent placement	5 (10.6%)	4 (13.8%)	1 (5.6%)	0.64
Final revascularization efficacy				
Complete	30 (63.8%)	20 (69.0%)	10 (55.6%)	
Partial	12 (25.5%)	6 (20.7%)	6 (33.3%)	
No	5 (10.6%)	3 (10.3%)	2 (11.1%)	0.67
Procedure-related complications				
Dissection	2 (4.3%)	2 (6.9%)	0	0.52
New distal embolization	4 (8.5%)	3 (10.3%)	1 (5.5%)	1.00
Bleeding events	2 (4.3%)	0	2 (11.1%)	0.14
Bowel resection	6 (12.8%)	3 (10.3%)	3 (16.7%)	0.66
Second-look laparotomy	7 (14.9%)	4 (13.8%)	3 (16.7%)	1.00
Length of hospital stay—day	7.0 (2.0–11.0)	7.0 (2.0–9.5)	8.5 (2.8–14.8)	0.14
In-hospital mortality	5 (10.6%)	4 (13.8%)	1 (5.6%)	0.64
30-day mortality	15 (31.9%)	9 (31.0%)	6 (33.3%)	0.87
Short bowel syndrome	1 (2.1%)	0	1 (5.5%)	0.38

Data are presented as *n* (%) or median (interquartile range) unless otherwise stated.

PMT, percutaneous mechanical thrombectomy; CDT, catheter-directed thrombolysis; AMI, acute mesenteric ischemia; PTA, percutaneous transluminal angioplasty.

**Figure 2:**
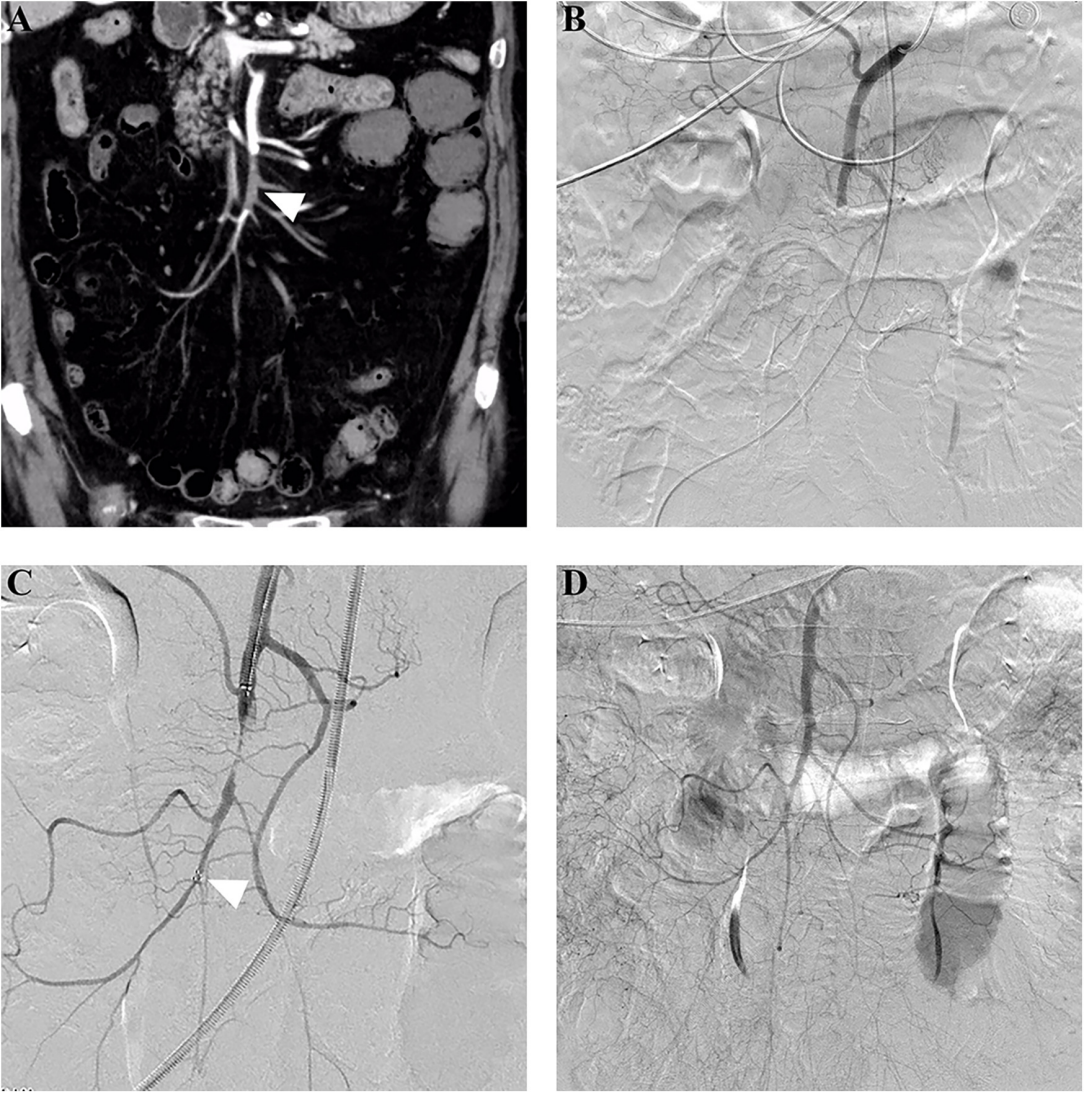
Successful stent thrombectomy in a patient with acute embolic SMA occlusion. **(A)** An 80-year-old woman presented with acute abdominal pain for 24 h, and subsequent computed tomography angiography revealed embolic occlusion (arrowhead) in the middle SMA stem. **(B)** Pre-procedure angiography further confirmed the occlusion without distal SMA stem and branch visualization. **(C)** Stent thrombectomy using a 6 mm × 30 mm Solitaire AB stent was performed. The arrowhead indicated the distal mark of the stent. **(D)** Post-procedure angiography showed complete SMA revascularization after one thrombectomy attempt. SMA,superior mesenteric artery.

**Figure 3:**
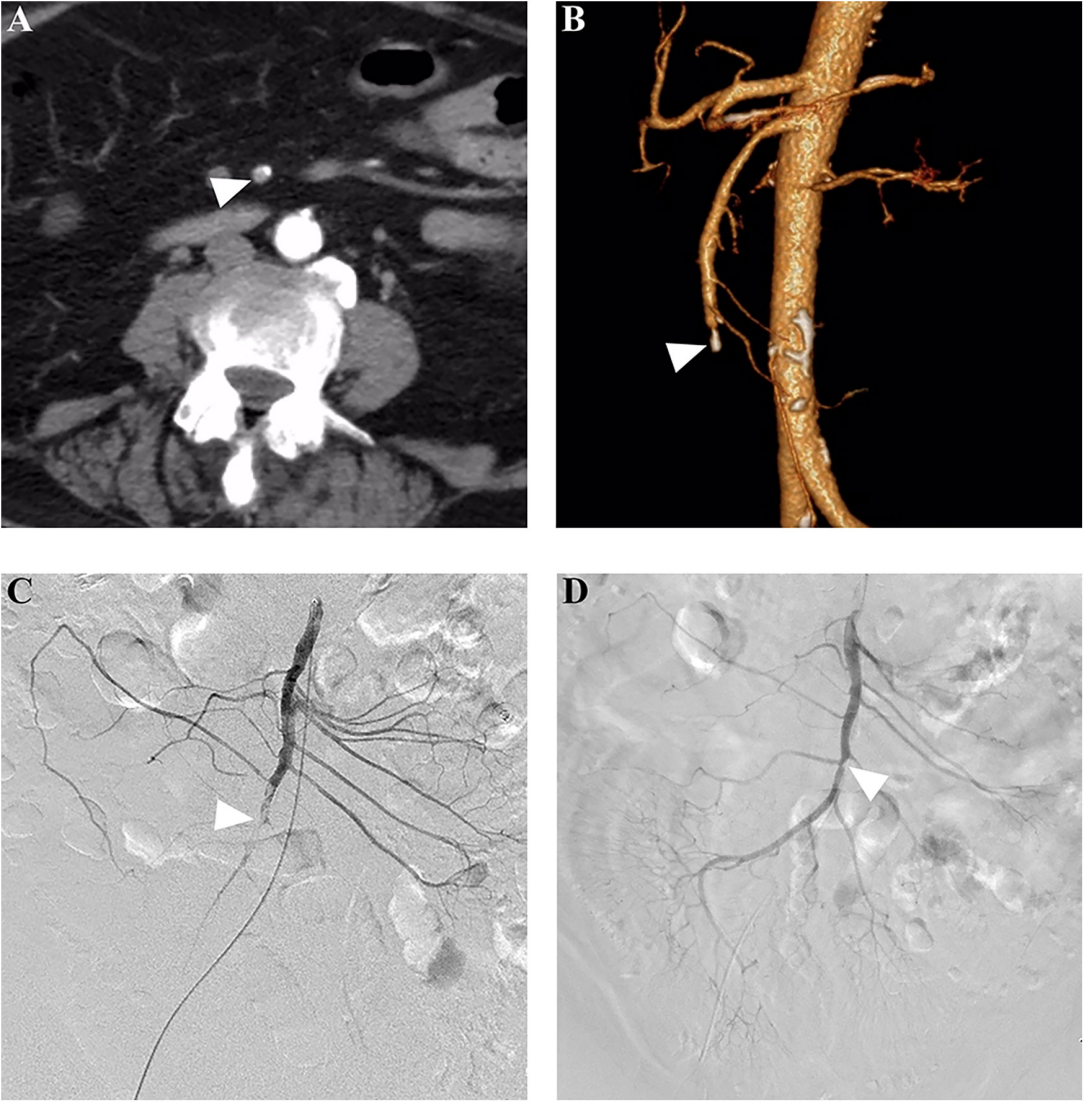
Successful catheter-directed thrombolysis in a patient with acute thrombotic SMA occlusion. **(A)** A 72-year-old woman presented with acute abdominal pain for 24 h, and subsequent computed tomography angiography revealed a filling defect (arrowhead) in the SMA stem. **(B)** The three-dimensional reconstruction showed distal SMA stem occlusion and calcification (arrowhead) in the proximal segment of the occluded artery. **(C)** Pre-procedure angiography showed a filling defect (arrowhead), and an end-hole catheter was placed for local thrombolysis using rt-PA at a rate of 10 mg/24 h. **(D)** After a thrombolysis duration of 2.0 days, complete revascularization was achieved, and the arrowhead showed a slight stenosis caused by the pre-existing calcification. SMA, superior mesenteric artery.

For patients who underwent PMT as the first EVR technique, initial completed, partial, and unsuccessful revascularization was achieved in 55.2% (16/29), 24.1% (7/29), and 20.7% (6/29) of patients, respectively, and adjunctive local thrombolysis was performed in 24.1% (7/29) of patients. After all adjunctive procedures, completed and partial revascularization was achieved in 69.0% (20/29) and 20.7% (6/29) of patients, respectively. Figure 2 shows a successful PMT procedure without adjunctive procedures. For patients who underwent CDT as the first EVR technique, initial completed, partial, and unsuccessful revascularization was achieved in 50% (9/18), 33.3% (6/18), and 16.7% (3/18) of patients, respectively. After adjunctive procedures, completed and partial revascularization was achieved in 55.6% (10/18) and 33.3% (6/18) of patients, respectively. Figure 3 shows a successful CDT procedure.

There was no significant difference regarding PTA/stent placement, revascularization rate, procedure-related complications, bowel resection, second-look laparotomy, short-term mortality, or short bowel syndrome between PMT and CDT as the first EVR technique (Table 2).

### Risk factors for 30-day mortality

No significant difference regarding the quality of revascularization between survivors and nonsurvivors was noted (Table 3). The univariable analysis identified significant differences in plasma lactate (1.5 mmol/L vs. 3.4 mmol/L, *p* = .038) and D-dimer (2.8 mg/L vs. 6.4 mg/L, *p* = .002) levels between survivor and nonsurvivor at 30 days (Table 3). Univariable logistic regression analysis found that plasma lactate and D-dimer levels were associated with an increased 30-day mortality (Table 4). Moreover, the significant association of plasma lactate (adjusted OR: 1.73; 95% CI: 1.13–2.66; *p* = .012) and D-dimer (adjusted OR: 1.73; 95% CI: 1.20–2.50; *p* = .003) remained in the multivariable logistic regression adjusted for age, female, and symptom duration (Table 4).

**Table 3:** Comparison of the potential risk factors for 30-day mortality between survivor and nonsurvivor.

Variable	Survivor (*n* = 32)	Nonsurvivor (*n* = 15)	*p* value
Demographics			
Mean age—year	75.0 ± 8.2	72.9 ± 6.3	0.19
Female	21 (65.6%)	10 (66.7%)	0.94
Etiology			
Thrombotic	6 (18.8%)	3 (20.0%)	
Embolic	26 (81.3%)	12 (80.0%)	1.00
Symptom to admission—hour	12.0 (6.0–24.0)	18.0 (10.0–24.0)	0.34
Manifestations			
Hematochezia	8 (25.0%)	7 (46.7%)	0.18
Peritonitis	2 (6.3%)	1 (6.7%)	1.00
Comorbidities			
Hypertension	19 (59.4%)	11 (73.3%)	0.30
Diabetes	7 (21.9%)	4 (26.7%)	0.72
Atrial fibrillation	18 (56.3%)	10 (66.7%)	0.50
Rheumatic heart disease	3 (9.4%)	0	0.54
Coronary artery diseases	10 (31.3%)	1 (6.7%)	0.08
Heart failure	6 (18.8%)	1 (6.7%)	0.40
Ischemic stroke	11 (34.4%)	2 (13.3%)	0.18
Chronic renal insufficiency	3 (9.4%)	2 (13.3%)	0.65
Pre-procedure laboratory values			
White blood cell count—10^9^/L	15.3 (11.0–17.2)	14.8 (9.2–20.9)	0.77
Plasma creatinine—μmol/L	82.1 (61.6–110.3)	112.0 (84.0–141.2)	0.073
Plasma lactate—mmol/L	1.5 (1.2–2.7)	3.4 (1.2–6.7)	0.038
D-dimer—mg/L	2.8 (1.4–3.6)	6.4 (2.6–12.3)	0.002
Image findings			
SMA branch involvement	3 (9.4%)	3 (20.0%)	0.37
Synchronous embolization	2 (6.3%)	1 (6.7%)	1.00
Evidence of bowel necrosis	2 (6.3%)	3 (20.0%)	0.31
Successful revascularization	30 (93.8%)	12 (80.0%)	0.31
Complete revascularization	23 (71.9%)	7 (46.7%)	0.094

Data are presented with *n* (%) or median (interquartile range) unless otherwise stated.

SMA, superior mesenteric artery; CI, confidence interval.

**Table 4:** Logistic regression models for the association between potential risk factors and 30-day mortality.

Variables	Model 1			Model 2		
	OR	95% CI	*p*-value	Adjusted OR	95% CI	*p*-value
Plasma lactate	1.33	1.04–1.71	0.022	1.73	1.13–2.66	0.012
D-dimer	1.45	1.12–1.88	0.005	1.73	1.20–2.50	0.003
Age				0.90	0.79–1.02	0.11
Female				3.12	0.35–27.93	0.31
Symptom duration				1.00	0.98–1.03	1.00

Model 1 is unadjusted. Model 2 is adjusted for age, sex, and symptom duration.

OR, odds ratio; CI, confidence interval.

## Discussion

The present study found that both PMT and CDT for the treatment of arterial AMI were associated with high successful revascularization and low complication rates. There was no significant difference in clinical outcomes between PMT and CDT. Elevated plasma lactate and D-dimer values were associated with increased 30-day mortality risk.

Arterial AMI remains a huge therapeutic challenge regardless of the increasingly advocated EVR first strategy (9, 22). In the recently published prospective multicenter AMESI study, the 30-day mortality for arterial AMI remained nearly 50% (2). Despite recent studies showing no significant difference regarding short-term mortality between EVR and OSR (5, 23), EVR first strategy is still preferred in patients with frail status or high morbidity burden (17).

Limited comparable evidence regarding endovascular treatment was available (11). CDT is a simple EVR technique, and it is associated with a satisfactory success rate and few complications (24, 25). In the present study, initial successful thrombolysis was achieved in 83.3% of patients, which is consistent with the previous publication (24). CDT is largely limited by a prolonged revascularization time, increased bleeding risk (24), and possible worsening during non-effective thrombolysis. As such, guidelines did not recommend CDT for patients with peritonitis and high bleeding risk (17).

Compared with CDT, PMT can promptly restore the compromised blood flow, thus potentially improving clinical outcomes. Manual aspiration using a large bore catheter is a simple and feasible PMT modality. Although the reported success rate was satisfactory (73.3%–100%) (26, 27), blood loss during aspiration may be a considerable issue (27). Moreover, the absence of revascularization after several passes should be considered a failure, and adjunctive thrombolysis or other PMT techniques may be needed. Stent thrombectomy is an important treatment modality for acute ischemic stroke with large vessel occlusion (28). This technique has been introduced to treat arterial AMI and achieved a satisfactory success rate (12). Previous studies suggested that Rotarex thrombectomy is associated with high technical success (14) and a satisfactory recanalization rate (14, 29). However, procedure-related complications, including vessel perforations and catheter tip fracture, have been reported (14, 29). Moreover, the rationale for Rotarex devices is thrombus fragmentation in combination with suction, which may be associated with the risk of distal embolization (29). The overall final successful revascularization rate for PMT in the present study was 89.7%, which is comparable to a previous study (29). The high successful revascularization rate in the present study may be associated with sufficient adjunctive therapies to address residual thrombus or pre-existing stenosis.

Few studies compared the outcomes between different EVR techniques. In a retrospective study including 31 patients, mechanical aspiration using the Indigo system (Penumbra, Inc., USA) failed to provide better results than manual aspiration (30). The present study compared PMT vs. CDT as the first EVR technique for arterial AMI, and no significant differences in clinical outcomes were observed. Unfortunately, the present study was unable to perform device-specific subgroup analyses due to the retrospective design and small sample size. Of note, the median symptom duration in the PMT group was significantly lower than in the CDT group, which may suggest that patients in the CDT group have less severe ischemia and are more able to tolerate thrombolysis.

Unfortunately, the present study failed to identify the association between successful revascularization and mortality, which may be attributed to the insufficient power of the study. But even in a larger study, the association between quality of revascularization and outcomes has not been shown (22). Various predictive factors for poor prognosis of AMI have been reported, such as advanced age (23, 31, 32), higher comorbidity burden (32), high C-reactive protein (22), and decreased bowel enhancement (22). The present study identified elevated plasma lactate and D-dimer values were associated with increased 30-day mortality. Elevated lactate levels are often associated with various severe pathological conditions such as sepsis and multiple organ dysfunction syndrome, which may lead to high mortality rates (33). The elevated D-dimer may be associated with a prolonged ischemia time (34) and more extensive thrombus, which may also lead to a poor prognosis. Patients with elevated lactate and elevated D-dimer levels may have advanced ischemia, and OSR should be considered in these patients to promptly restore blood flow and directly evaluate the bowel gangrene.

Some important limitations to the present study should be noticed. First, this was a single-center retrospective study with a limited sample size in a relatively long study period. Selection bias and other confounding factors inherent to the study design may have biased the results. For instance, patients had severe symptoms may have received PMT, and patients with milder symptoms may have received CDT. Additionally, a small cohort inherently restricts statistical power, particularly in detecting subtle differences between PMT and CDT. Second, three different techniques were classified as PMT, which might have influenced the results since the efficacy may vary among the different techniques. Third, the adjunctive use of thrombolysis and stent placement could bias an evaluation of the effect of PMT or CDT as standalone.

In conclusion, PMT and CDT were associated with a high successful revascularization rate and a low complication rate in the treatment of arterial occlusive AMI. No significant clinical differences between PMT and CDT were noted. Elevated plasma lactate and D-dimer values may be associated with increased 30-day mortality.

## Data Availability

The original contributions presented in the study are included in the article/Supplementary Material, further inquiries can be directed to the corresponding author.
